# Supplementary material for: Identifying domains of applicability of machine learning models for materials science
Source: Nat Commun. 2020 Sep 4;11:4428. doi: 10.1038/s41467-020-17112-9 (PMC7474068; doi:10.1038/s41467-020-17112-9)
Supplement: Supplementary file 1 — Supplementary Information [file 41467_2020_17112_MOESM1_ESM.pdf]

Supplementary Information for:
Identifying Domains of Applicability of Machine Learning Models for Materials Science

Christopher Sutton, Mario Boley, Luca M Ghiringhelli, Matthias Rupp, Jilles Vreeken, Matthias Scheffler

Supplementary Notes

Supplementary Note 1

The main text discusses the DA selectors that are identified most often through the six fold cross-validation. See Supplementary Table 1 for a detailed list of selectors encountered in the individual splits. Indeed, the identified selectors are mostly stable, i.e., appearing in four out of six splits for MBTR and SOAP and five out of six for n-gram. Note that, in case of MBTR, the first two selectors are virtually equivalent with a minor variation in the threshold on the γ angle. Generally, for all models the conditions of the alternative selectors have high overlap with the conditions of the majority selector. This also holds for the atomic property model, for which essentially equivalent selectors are identified in four out of six splits. For completeness, Supplementary Figures 1 and 2 show the distributional details of the variables of the MBTR and n-gram selectors presented in the main text.

Supplementary Table 1. List of DA selectors identified on different DA identification / DA validation set splits.

model	selector	splits
MBTR	$N \geq 50$ atoms $\wedge \gamma \leq 98.8° \wedge R_{\text{Al-O}} \leq 2.1$ Å	3
	$N \geq 50$ atoms $\wedge \gamma \leq 93.8° \wedge R_{\text{Al-O}} \leq 2.1$ Å	1
	$b \geq 5.6$ Å $\wedge a/c \leq 4.0 \wedge \gamma < 90.4° \wedge R_{\text{Al-O}} \leq 2.1$ Å	1
	$N \geq 50$ atoms $\wedge \gamma \leq 98.8°$	1
SOAP	$a/c \leq 3.9 \wedge \gamma < 90.4° \wedge \beta \geq 88.7°$	4
	$a/c \leq 3.9 \wedge \alpha < 61.0° \wedge \beta \geq 88.7° \wedge R_{\text{Al-O}} \leq 2.1$ Å	1
	$c \geq 4.1$ Å $\wedge \alpha \geq 61.0° \wedge \gamma \leq 92.2° \wedge \beta \geq 88.7°$	1
n-gram	$b \geq 5.6$ Å $\wedge \gamma < 90.4° \wedge R_{\text{Al-O}} \leq 2.1$ Å $\wedge R_{\text{Ga-O}} \leq 2.1$ Å	5
	$\alpha \geq 61.0° \wedge b \geq 5.6$ Å $\wedge R_{\text{Al-O}} \leq 2.1$ Å $\wedge R_{\text{Ga-O}} \leq 2.1$ Å	1
atomic	$a/c \leq 3.9 \wedge \gamma \leq 98.8°$	2
	$a/c \leq 3.9 \wedge \gamma \leq 99.0°$	1
	$a/c \leq 3.9 \wedge \gamma \leq 98.5°$	1
	$a/c \leq 3.9 \wedge \gamma \leq 98.8° \wedge b \geq 5.6$ Å	1
	$a/c \leq 3.9 \wedge \gamma \leq 98.8° \wedge b \geq 5.6$ Å $\wedge R_{\text{Ga-O}} \leq 2.1$ Å	1

Supplementary Note 2

In the main text we investigated the DAs resulting from optimizing the effect on the relative model error. This was to obtain an SGD objective and by extension DA selectors that are uncorrelated with the prediction target variable. In contrast, the absolute error is not guaranteed to be uncorrelated from the target: for instance, for our investigated models, we observe empirical Pearson product moment correlation coefficients on the test set of 0.37 (MBTR), 0.38 (SOAP), 0.33 (n-gram), and 0.4 (atomic). Thus using the absolute error for DA identification is likely to also result in correlated DA selectors, which is undesired for our purpose of uncovering characteristic distinctions in the performance of the different representations. When using the DAs in rapid screening scenarios we are less concerned with them having a higher overlap and we thus can directly optimize the absolute error. See Supplementary Figure 3 for a visualization of the absolute error distributions resulting from this approach and Supplementary Table 2 for the numerical summary statistics.

Indeed, the achieved error reductions are slightly higher using this objective function than for using the relative error. On the other hand, the DAs also confirm the anticipated issues of having a less representative distribution of the prediction target (e.g., for SOAP where DAs of similar size are identified with both objective functions we have a target standard deviations of 91.6 for the absolute error DA compared to a target standard deviation of 97.8 for the relative error DA, which is closer to the global standard deviation of 106.5). Moreover, looking at the identified selectors (see Supplementary Table 3), we observe that this correlation with the formation energy indeed also reduces the differences between the DAs of the different models, which now have more overlapping components. In fact, for MBTR and SOAP we end up with the same DA in four and five, respectively, out of six splits in the cross validation.

Supplementary Note 3

Supplementary Table 4 contains a list of atomic properties used for the baseline model discussed in the main text. Feature values for a compositions are obtained by forming a weighted average of the property values according to the mixture coefficients.

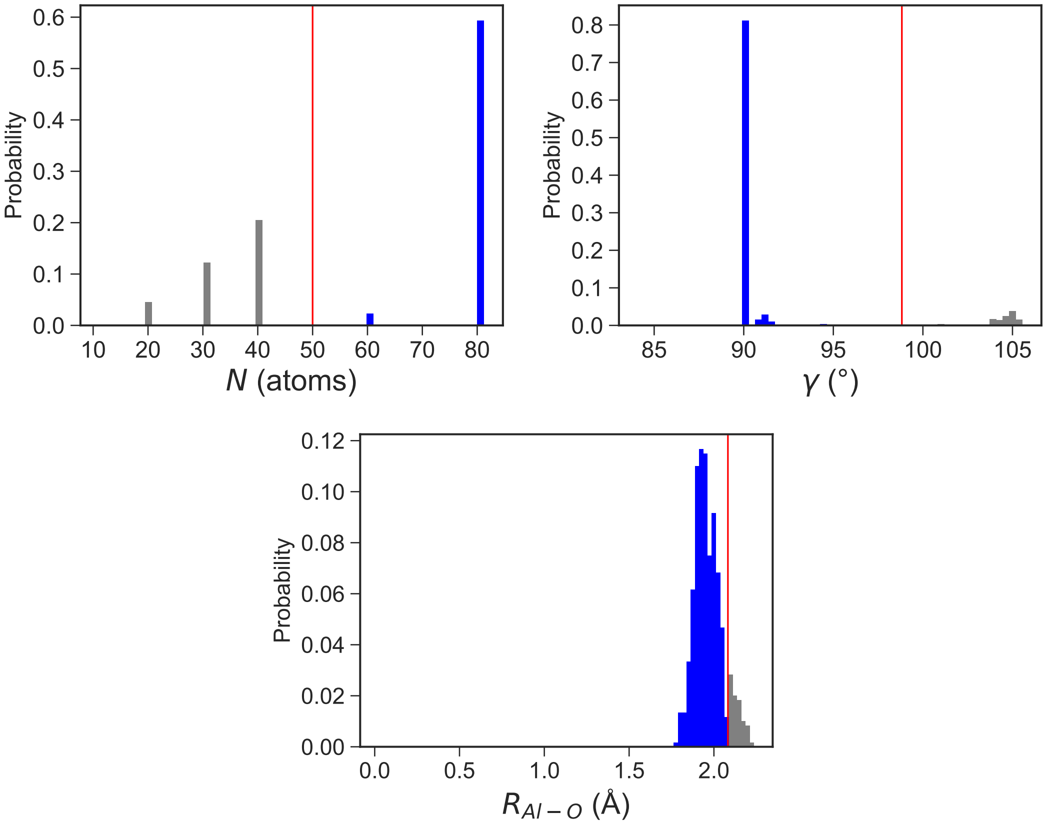

Supplementary Figure 1. Effect of individual DA selector conditions for MBTR-based model. The distributions of the three features referenced in the selector $\sigma_{\text{MBTR}} \equiv N \geq 50$ atoms $\wedge \gamma \leq 98.8° \wedge R_{\text{Al-O}} \leq 2.1$ Å, i.e., N (**top left**), γ (**top right**), and $R_{\text{Al-O}}$ (**bottom**), are shown with sub-population selected by condition in blue, sub-population deselected in gray, and threshold by red line.

Supplementary Table 2. Summary statistics for DAs resulting from optimizing *absolute error*. Coverage (cov), mean absolute error (MAE), 95-percentile absolute error (95AE), and coefficient of determination based on absolute error (R) are all estimated via mean value from non-overlapping splits of the test set into DA validation set (100-values) and DA identification set (500-values). Standard deviations are in parentheses. Global values are computed over whole test set. MAE and 95AE are in units of meV/cation, cov values are in percentages.

	Global (test set)			DA (validation set)				DA (identification set)			
	MAE	95AE	R	cov	MAE	95AE	R	cov	MAE	95AE	R
MBTR	14.2	54.1	0.83	65 (3)	8.9 (0.9)	26.5 (5.0)	0.88 (0.01)	64 (1)	8.3 (0.2)	27.3 (0.4)	0.89 (0.00)
SOAP	14.1	51.0	0.84	65 (3)	8.6 (1.0)	28.4 (5.5)	0.89 (0.01)	64 (1)	8.0 (0.2)	23.4 (0.4)	0.89 (0.00)
n-gram	14.7	51.1	0.83	66 (2)	11.3 (0.5)	33.6 (4.4)	0.84 (0.01)	69 (1)	9.3 (0.1)	30.0 (1.5)	0.88 (0.00)
atomic	65.5	154.5	0.24	43 (4)	36.9 (2.0)	84.5 (7.3)	0.39 (0.02)	42 (0)	36.6 (0.7)	89.2 (1.9)	0.43 (0.00)

Supplementary Note 4

The effect term of the impact objective function captures a reduction in error relative to the global error (see Equation (4) and Supplementary Equation (1) below). This calibrates the objective function, but it only guarantees that a positive objective value corresponds to an “increased” applicability as opposed to categorical applicability in terms of any absolute error constraint. Hence, the method detects the best DA possible out of all candidates, but in extreme cases no (notable) improvement over the global domain might be possible.

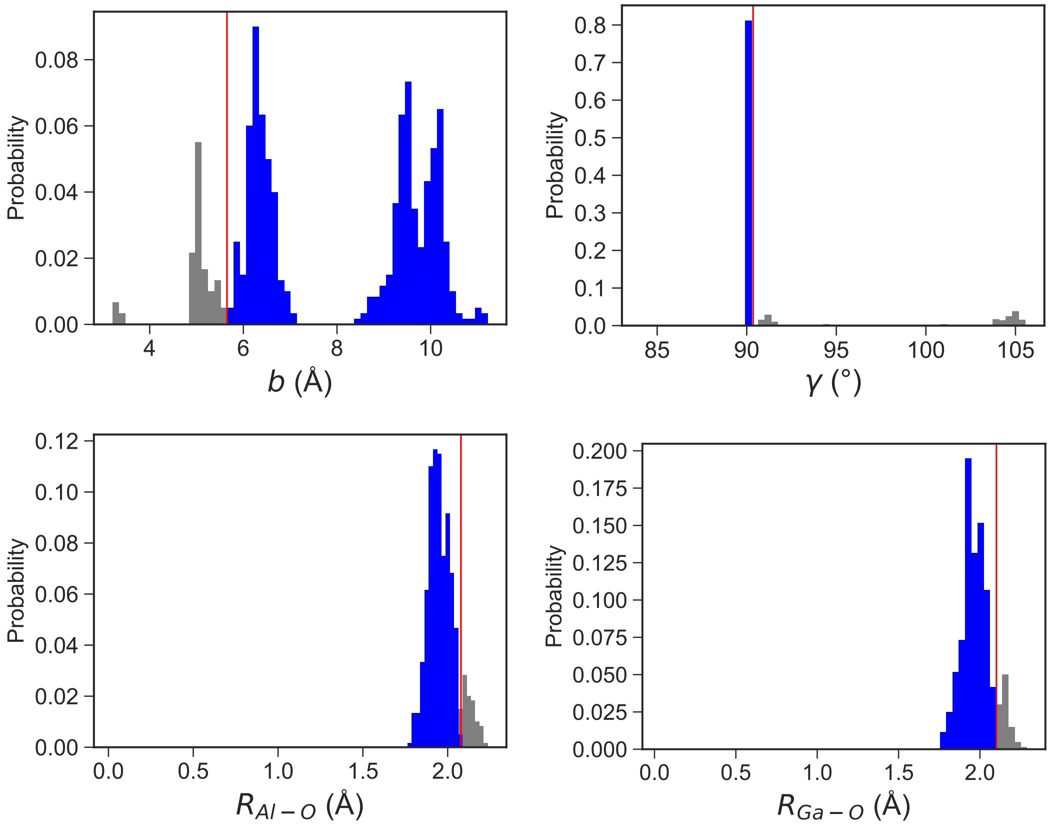

Supplementary Figure 2. Effect of individual DA selector conditions for n-gram-based model. The distributions of the four features referenced in the selector $\sigma_{\text{n-gram}} \equiv \; b \geq 5.6\,\text{Å} \wedge \gamma < 90.4° \wedge R_{\text{Al-O}} \leq 2.1\,\text{Å} \wedge R_{\text{Ga-O}} \leq 2.1\,\text{Å}$, i.e., b (**top left**), γ (**top right**), $R_{\text{Al-O}}$ (**bottom left**), and $R_{\text{Ga-O}}$ (**bottom right**), are shown with sub-population selected by condition in blue, sub-population deselected in gray, and threshold by red line.

Supplementary Table 3. List of DA selectors identified when optimizing the *absolute error*.

model	selector	splits
MBTR	$\alpha \geq 61.0° \wedge R_{\text{Al-O}} \leq 2.1\,\text{Å} \wedge \beta \geq 88.7° \wedge \%\text{Ga} \geq 0.089$	5
	$N \geq 50$ atoms	1
SOAP	$\alpha \geq 61.0° \wedge R_{\text{Al-O}} \leq 2.1\,\text{Å} \wedge \beta \geq 88.7° \wedge \%\text{Ga} \geq 0.089$	4
	$\alpha \geq 61.0° \wedge R_{\text{Ga-O}} \geq 0.88\,\text{Å} \wedge R_{\text{In-Al}} \leq 3.2\,\text{Å}$	2
n-gram	$\alpha \geq 61.0° \wedge R_{\text{Al-O}} \leq 2.1\,\text{Å} \wedge R_{\text{Ga-O}} \in [0.88\,\text{Å}, 2.1\,\text{Å}]$	3
	$\alpha \geq 61.0° \wedge b \geq 5.6\,\text{Å} \wedge R_{\text{Al-O}} \leq 2.1\,\text{Å} \wedge R_{\text{Ga-O}} \leq 2.1\,\text{Å}$	1
	$\alpha \in [61.0°, 119°] \wedge R_{\text{Al-O}} \leq 2.1\,\text{Å} \wedge R_{\text{Ga-O}} \leq 2.1\,\text{Å}$	1
	$\alpha \in [61.0°, 119°] \wedge b \geq 5.6\,\text{Å} \wedge R_{\text{Ga-O}} \leq 2.1\,\text{Å}$	1
atomic	$a \leq 13.7\,\text{Å} \wedge a/c \leq 3.9 \wedge a/b \leq 3.2 \wedge V/N \leq 12.6\,\text{Å}^3$	1
	$a \leq 13.8\,\text{Å} \wedge a/c \leq 3.9 \wedge a/b \leq 3.2 \wedge V/N \leq 12.6\,\text{Å}^3$	1
	$a \leq 13.8\,\text{Å} \wedge a/c \leq 3.9 \wedge a/b \leq 3.2 \wedge V/N \leq 12.7\,\text{Å}^3$	1
	$a \leq 13.8\,\text{Å} \wedge a/b \leq 3.2 \wedge V/N \leq 12.6\,\text{Å}^3$	1
	$a \leq 14.3\,\text{Å} \wedge a/c \leq 3.2 \wedge V/N \leq 12.7\,\text{Å}^3 \wedge \alpha \geq 61.0°$	1
	$a \leq 13.8\,\text{Å} \wedge a/c \leq 3.2 \wedge V/N \leq 12.8\,\text{Å}^3$	1

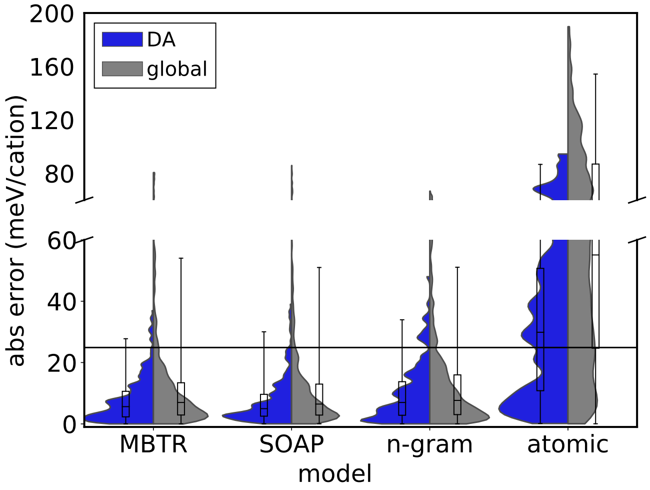

Supplementary Figure 3. DA versus global model performance for DAs identified by minimizing the absolute error. Comparison of absolute error distributions for entire test set (global) and for DA validation sets (DA). Violin plots extend to the 98% percentile. Boxplots inside violin plots indicate 25%, 50%, and 75% percentiles of the absolute errors, as well as 5% and 95% percentiles (whiskers). Horizontal line indicates reference error level of half of the mean energy difference between the minimum energy and the second-to-minimum energy polymorph (mean over all considered concentrations).

Supplementary Table 4. Atomic properties used for baseline model discussed in the main text.

Electron affinity
Ionization potential
Highest-occupied atomic orbital
Lowest unoccupied atomic orbital
Atomic radii from Rahm et al.
Covalent atomic radii
Free-atom *d* radius
Free-atom *p* radius
Free-atom *s* radius
Heats of formation
Atomic mass
Volume of the ICSD entry for Al_2O_3, Ga_2O_3, and In_2O_3

Supplementary Methods

Coverage/Effect Trade-off

As indicated in the main text, a sensible DA objective function takes into account both coverage and effect (on model error), in a non-compensatory way. This means that a DA with close to zero error reduction should have close to zero objective value even if the coverage value is very close to 1 (similar the other way around). Additionally, we require the objective to be smooth and monotonous in both criteria. That is, if we write our objective function in the form $f(\sigma) = g(\mathrm{cov}(\sigma), \mathrm{eff}(\sigma))$ we require for $x_0, y_0, \varepsilon > 0$ that

(i) g is smooth in both arguments,

(ii) $g(x_0 + \varepsilon) > g(x_0, y_0)$ and $g(x_0, y_0 + \varepsilon) > g(x_0, y_0)$,

(iii) $\lim_{x\to 0} g(x, y_0) = \lim_{y\to 0} g(x_0, y) = 0$.

In particular, requirement (ii) assures that the DA optimization will never yield selectors that are not Pareto-optimal with respect to the underlying bi-criterial optimization problem. This means that the resulting selector will never be dominated by another selector in terms of both, coverage and effect.

The impact function presented in the main text satisfies all these criteria. At the same time, it weighs coverage and effect equally, and there are application contexts where the relative importance of the two criteria is not uniform. A simply way to allow for a non-equal numerical weighting while retaining the above properties is to augment the impact function by a numerical trade-off parameter $\gamma \in (0,1)$ as follows:

$$\mathrm{impact}(\sigma) = \underbrace{\left(\frac{s}{k}\right)^{\gamma}}_{\text{coverage}} \underbrace{\left(\frac{1}{k}\sum_{i=1}^{k} l_i(f) - \frac{1}{s}\sum_{i \in I(\sigma)} l_i(f)\right)_{+}^{1-\gamma}}_{\text{effect on test error}} \tag{1}$$

where by using $(\cdot)_+ = \max\{0,\cdot\}$ we cap the effect part from below by 0. By varying γ over $(0,1)$ we can find DAs that realize all Pareto-optimal trade-offs between coverage and effect.

See Supplementary Figure 4 for an illustration of the range of DAs that can be identified for the evaluated ML models by varying the trade-off parameter. We can see that the SOAP model does in fact allow for a DA that matches the error and and coverage of the MBTR DA discussed in the main text, but, putting more weight on coverage per default, this DA was ignored in favor of the reported one was larger coverage.

Note that common subgroup discovery implementations offer an alternative parameterization of the impact function that only uses one exponent $\gamma' \in (0,\infty)$ on the coverage term. These two parameterizations are equivalent in the sense that we can find the same optima by using the transformations $\gamma = \gamma'/(1+\gamma')$ and $\gamma' = \gamma/(1-\gamma)$ to translate between both versions. It is important to note though that a uniform search over the coverage/effect trade-off space is most easily achieved by the first parameterization.

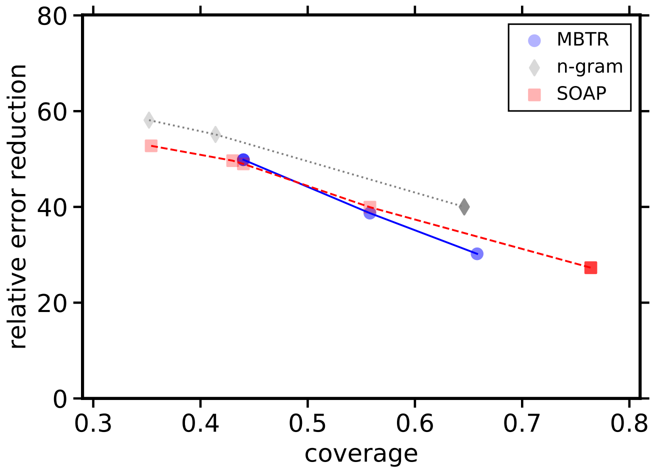

Supplementary Figure 4. Coverage/effect trade-off. Coverage versus relative error reduction of the DAs for the various models achieved by varying over the trade-off parameter γ of the augmented impact function given in Supplementary Equation (1) for 8 values from 0.25 and 2.0 in steps of 0.25.

Algorithms and Computational Complexity

Optimizing the subgroup discovery objective function over the full set of conjunctive selectors is in fact an NP-hard combinatorial optimization problem (where the input size is taken as the number of base propositions and the number of data points). Thus, we cannot hope for a worst-case polynomial time algorithm. However, a practically effective branch-and-bound algorithm exists for the objective function.

This algorithm recursively generates a part of the set of possible subgroup selectors (branching operator) but does not expand selectors that cannot lead to an improvement of the currently best selector based on an upper bound of attainable objective values (bounding function). In particular, the branching operator makes sure to generate exactly one representative of every equivalence class of selectors[1] (we call two selectors σ and σ' equivalent if they select they same data points, i.e., $I(\sigma) = I(\sigma')$) and generates them in a general-to-specific order (i.e., if σ' is generated from σ then $I(\sigma) \supset I(\sigma')$).

Based on the second property, an effective and efficiently computable bounding function can be defined by considering the impact value attained by a hypothetical specialization σ' of a given selector σ that precisely describes all data points selected by σ that have a below-average loss value. Formally:

$$\begin{aligned} b(\sigma) &= \max\left\{ \frac{|J|}{k} \left(\sum_{i=1}^{k} \frac{l_i(f)}{k} - \sum_{i \in J} \frac{l_i(f)}{|J|} \right) : J \subseteq I(\sigma) \right\} \\ &= \frac{|I_-(\sigma)|}{k} \left(\sum_{i=1}^{k} \frac{l_i(f)}{k} - \sum_{i \in I_-(\sigma)} \frac{l_i(f)}{|I_-(\sigma)|} \right) \end{aligned}$$

where we denote by $I_-(\sigma)$ the subset of indices selected by σ that have a below-average loss value and by $|S|$ the cardinality of a set S. The first equality shows that all selectors more specific than σ cannot have an objective value greater than $b(\sigma)$, which makes b an admissible bounding function, and the second equation shows how b can be efficiently computed (slightly more complicated variants[2,3] are needed when we use the non-uniform coverage/effect trade-off discussed in Supplementary Method *Coverage/Effect Trade-off*).

With this approach, SGD needs to explore in practice only a small fraction of the large combinatorial space of all possible subgroup selectors. In fact, the DA optimization is much faster than the KRR algorithm itself (despite the latter being worst-case polynomial). This is because the KRR parameter vector $\boldsymbol{\nu}$ is found via

$$\boldsymbol{\nu} = (\lambda I + K)^{-1}\mathbf{y}$$

involving the regularized inverse of the kernel matrix K with entries $K_{i,j} = \exp(-\|\boldsymbol{x}_i^F - \boldsymbol{x}_j^F\|^2/(2\varepsilon^2))$. Even if efficient approximation techniques like the Nyström method[4] are used, this operation tends to scale super-quadratic with the number of training data points n.

This is illustrated by the three KRR models fitted for this study. Running the SGD algorithm on our test set of 600 examples and 312 base propositions takes 11 seconds on a current personal computer (Intel(R) Xeon(R) Gold 6126 CPU 2.60GHz running GNU/Linux). In contrast, computing the n-gram representation on the 2400 training examples takes approximately 2 minutes, and computing the corresponding KRR model takes 10 minutes using `scikit-learn`.

Supplementary References

1. Boley, M. & Grosskreutz, H. Non-redundant subgroup discovery using a closure system. In *Joint European Conference on Machine Learning and Knowledge Discovery in Databases*, 179–194 (Springer, 2009).
2. Lemmerich, F., Atzmueller, M. & Puppe, F. Fast exhaustive subgroup discovery with numerical target concepts. *Data Min. Knowl. Discov.* **30**, 711–762 (2016).
3. Boley, M., Goldsmith, B. R., Ghiringhelli, L. M. & Vreeken, J. Identifying consistent statements about numerical data with dispersion-corrected subgroup discovery. *Data Min. Knowl. Discov.* **31**, 1391–1418 (2017).
4. Williams, C. K. & Seeger, M. Using the nyström method to speed up kernel machines. In *Advances in neural information processing systems*, 682–688 (2001).
